# Utilizing cardiac magnetic resonance to assess the sequelae of prior rejection episodes on myocardium and correlation with clinical outcomes

**DOI:** 10.1016/j.jhlto.2025.100298

**Published:** 2025-05-24

**Authors:** Akila Bersali, Farhan Ishaq, Mianli Xiao, Edward A Graviss, George Naufal, Ashrith Guha, Dipan J. Shah, Rayan Yousefzai

**Affiliations:** a Houston Methodist DeBakey Heart and Vascular Center, Division of Cardiovascular, Houston, TX; b Center for Health Data Science and Analytics Houston Methodist, Houston, TX

**Keywords:** Heart transplant, Cardiac MRI imaging, Acute cellular rejection, Rejection sequelae

## Abstract

This study compared structural and functional alterations using cardiac MRI (CMR) in heart transplant recipients with and without acute cellular rejection (ACR) and analyzed their association with clinical outcomes. ACR patients showed reduced left ventricular global longitudinal strain (LV GLS) (10% vs 12%; *P* = 0.03), reduced right ventricular global longitudinal strain (16% vs 18%; *P* = 0.04), increased left ventricular (LV) mass (72 vs 61 g/m²; *P* = 0.003), and decreased right ventricular stroke volume (70 vs 79 mL; *P* = 0.05). Univariate analysis revealed that LV ejection fraction (EF) (HR 0.90, *P* < 0.001), RV ejection fraction (HR 0.91, *P* = 0.004), LV stroke volume (SV) (HR 0.96, *P* = 0.01), RV SV (HR 0.96, *P* = 0.003), and LV scar size (HR 1.13, *P* = 0.002) were significantly associated with cardiovascular hospitalization or mortality. After adjusting for relevant covariates, indexed RV SV (HR 0.90, *P* = 0.015) and LV scar size (HR 1.17, *P* = 0.026) remained significant predictors of the clinical outcome. CMR can identify sequelae of ACR, potentially influencing clinical decisions.

## Background

Orthotopic heart transplantation remains a life-saving procedure for patients with end-stage heart failure.^1^ However, allograft rejection and dysfunction, both acute and chronic, continue to pose significant limitations, contributing to morbidity and mortality.^2^

Advances in cardiac magnetic resonance (CMR) imaging have provided insights into structural changes and ventricular remodeling in heart transplant recipients.^2^, ^3^, ^4^, ^5^, ^6^ This pilot study enrolled consecutive heart transplant recipients who underwent comprehensive CMR imaging. The objective was to evaluate functional and tissue characterization parameters to assess the lasting impact on the myocardium, even after treatment, stratified by prior acute cellular rejection (ACR) status. ACR has been widely recognized as a key factor influencing the long-term function of transplanted hearts. It is associated with a higher risk of graft dysfunction, increased mortality, and an increased likelihood of developing chronic rejection.^7^ Moreover, ACR can contribute to myocardial injury and adverse remodeling, potentially leading to diminished cardiac output and organ failure over time.^8^ Given these findings, our study aimed to investigate the lasting effects of ACR manifested in CMR parameters. We have used CMR over other imaging modalities due to its non-invasive nature and superior capabilities in myocardial tissue characterization, including the assessment of scar, inflammation, and injury. Additionally, the study explored correlations between these findings and clinical outcomes.

## Study population and methods

This study analyzed 70 consecutive heart transplant recipients who underwent CMR evaluations after transplantation between November 2016 and April 2023. Patients who underwent CMR within the first three months post-transplant were excluded due to the potential effects of transplant surgery on CMR parameters. Patients were stratified into two groups based on their prior history of ACR before the CMR. ACR was defined based on the International Society for Heart and Lung Transplantation grading system as grade 2R or higher. All patients diagnosed with ACR received treatment, and follow-up biopsies confirmed the resolution of rejection.

CMR was conducted using 1.5 T (Sola, Avento, and Aera) Siemens scanners with phased-array coils. The protocol included morphologic and functional assessment, T1 mapping using a 5(3)3 MOLLI before and 15-20 min post-contrast at the mid short-axis and a T2 mapping at the same location. Extracellular volume (ECV) and late gadolinium enhancement (LGE) imaging. Prior to inclusion, a rigorous quality assessment was performed, and no study was deemed unsuitable for analysis based on image quality.

LGE imaging was performed 10 min after contrast (0.15 mmol/kg) gadopentetate Level III reader performed CMR analysis. Precession software was used for functional assessment; scar size was calculated by summing weighted segmental scores over 16 segments.^9^ Tissue characterization and strain analysis (longitudinal strain) were done using cvi42 (v5.13 Circle Cardiovascular Imaging). Global T2, ECV, and longitudinal biventricular strain were assessed using the 16-segment AHA model. ECV values above 30% were considered elevated based on prior work from our institution.^10^ We adopted a T2 mapping cutoff of 50.5 ± 3.4 ms based on existing literature in heart transplantation.^11^

Descriptive statistics were presented as medians with interquartile ranges (IQR) for continuous variables and as frequencies with corresponding percentages for categorical variables. Group comparisons for categorical variables were performed using the chi-square test or Fisher’s exact test, as appropriate, while continuous variables were compared using the Wilcoxon rank-sum test. Receiver operating characteristic (ROC) curve analysis, with the Youden index, was employed to identify optimal cut-off values of CMR parameters for classifying ACR rejection. Kaplan-Meier survival curves were constructed to illustrate the cumulative incidence of the composite outcome (CV hospitalization and mortality), stratified by the ROC-derived cut-offs, with group differences assessed using the log-rank test. Univariable and multivariable Cox proportional hazards regression models were used to evaluate associations between clinical variables and the composite outcome. The multivariable model was adjusted for potential confounders, including age, sex, diabetes, hypertension, rejection treatment, ischemic time during transplant, and primary graft dysfunction (PGD). All statistical analyses were performed using R software (version 4.4.1; R Foundation for Statistical Computing, Vienna, Austria), and a two-tailed *p*-value < 0.05 was considered statistically significant.

## Results

Seventy heart transplant recipients who underwent CMR at a median of 5.9 months (IQR, 5.7-6.2 months) after transplantation between November 2016 and April 2023 were included in this study. The median time between the first episode of rejection and the CMR scan was 5.2 months (IQR 4.6-5.6 months). The baseline characteristics, including demographics, comorbidities, implantation, and use of immunosuppressants (shown in Table 1), were similar between patients with a history of ACR at any time before CMR and those without ACR. During the study period, all donors were from Donation After Brain Death, and ice storage was the only preservation method used. The treatment protocol for ACR remained the same throughout the study period. The functional and structural parameters assessed by CMR revealed several markers of early myocardial dysfunction in patients with a history of ACR. These markers included lower left ventricular global longitudinal strain (LV GLS) (10% IQR [8-13] vs 12% IQR [10-14]; *P* = 0.03) and right ventricular global longitudinal strain (RV GLS) (16% IQR [13-18] vs 18% IQR [16–20]; *P* = 0.04), as well as higher left ventricular (LV) mass (72 g/m² IQR [64-86] vs 61 g/m² IQR [58-70]; *P* = 0.003). The marker of functional dysfunction included a lower right ventricular stroke volume (RV SV) in patients with ACR history before the MRI scan (70 ml IQR [59-82] vs 79 ml IQR [68-93]; *P* = 0.05) (Table 1). No significant differences were found in other tissue characteristics between the two groups.

**Table 1:** Baseline Characteristics and CMR Parameters

	Total	No ACR	ACR	*P*-Values
	(*N* = 70)	(*N* = 43)	(*N* = 27)	
Demographics				
Age (years)	59 (51-66)	60 (55-66)	58 (43-66)	0.25
Male (%)	52 (74)	31 (72)	21 (78)	0.80
Race (%)				0.44
White or Caucasian	40 (57)	22 (51)	18 (67)	
Black or African American	23 (33)	16 (37)	7 (26)	
Others	7 (10)	5 (12)	2 (7)	
Comorbidities				
BMI	26 (23-29)	27 (24-29)	25 (22-28)	0.36
Diabetes (%)	37 (53)	24 (56)	13 (48)	0.71
PVD (%)	4 (6)	1 (2)	3 (11)	0.31
HTN (%)	59 (84)	35 (82)	24 (89)	0.62
History of CVA (%)	22 (31)	15 (35)	7 (26)	0.60
Previous Malignancy (%)	5 (7)	5 (12)	0 (0)	0.17
Heart-Kidney Transplant (%)	18 (25.7)	14 (32.6)	4 (14.8)	0.17
Heart-Liver Transplant (%)	5 (7.1)	4 (9.3)	1 (3.7)	0.68
Heart-Kidney-Liver Transplant	2 (2.9)	2 (4.7)	0 (0)	0.69
Immunosuppressants				
Tacrolimus (%)	66 (94)	39 (91)	27 (100)	0.27
Sirolimus (%)	2 (3)	1 (2)	1 (4)	1.00
Cyclosporine (%)	1 (1.4)	1 (2.3)	0 (0)	1.00
Implantation				
PGD (%)	14 (20.0%)	5 (11.6%)	9 (33.3%)	0.057
Ischemic Time (min)	228 (203-250)	227 (217-247)	229 (197-253)	0.751
CMR parameters				
Functional parameters				
LV GLS (%)	−11(9.6-13)	−12 (10-14)	−10 (8-13)	***0.03***
LV EDV (ml)	137 (112-154)	140 (114-151)	134 (109-156)	0.71
LV EDVI (ml/m²)	69 (61-80)	70 (63-78)	69 (57-82)	0.77
LV ESV (ml)	55 (42-73)	55 (46-70)	62(40-77)	0.87
LV ESVI (ml/m²)	29 (23-37)	29 (24-33)	30 (20-38)	0.82
LV mass (g)	123 (113-150)	121 (109-131)	138 (118-172)	***0.03***
Indexed LV mass (g/m²)	65 (59-75)	61 (58-70)	72 (64-86)	***0.003***
LV wall thickness (AS) (mm)	1 (0.8-1.1)	1 (0.9-1.1)	1 (0.9-1.2)	0.37
LV wall thickness (IS) (mm)	0.9 (0.7-1)	0.8 (0.7-1)	0.9 (0.8-1.1)	***0.04***
LV SVI (ml/m²)	40 (35-43)	39 (36-44)	40 (34-42)	0.38
LV EF (%)	57 (52-63)	57 (53-62)	57 (51-65)	0.97
RV GLS	−17 (15-20)	−18 (16-20)	−16(13-18)	***0.04***
RV EDV (ml)	150 (121-174)	154 (130-182)	146 (116-167)	0.18
RV EDVI (ml/m²)	77 (68-89)	79 (71-88)	71 (61-89)	0.31
RV ESV (ml)	75 (57-91)	76 (60-91)	68 (52-92)	0.61
RV ESVI (ml/m²)	38 (32-45)	38 (33-43)	38(26-46)	0.94
RV SV (ml)	75 (64-88)	79 (68-93)	70 (59-82)	***0.05***
RV SVI (ml/m²)	38 (34-43)	40(37-45)	38 (31-40)	0.06
RV EF (%)	51 (47-56)	51 (48-56)	51 (46-55)	0.45
Tissue Characterization				
Global T2 (msec)	47 (42-50)	46 (42-48)	47 (43-52)	0.21
Global ECV (%)	30 (28-34)	29 (27-32)	32 (29-35)	0.09
LV Scar size (%)	0 (0-2.1)	0 (0-1.5)	0 (0-3.8)	0.94

Values are in number (%) unless otherwise indicated.

ACR, acute cellular rejection; AS, antero-septal; BMI, body mass index; BSA, body surface area; CMR, cardiovascular magnetic resonance; CO, cardiac output; ECV, extra-cellular volume; EDD, end diastolic diameter; EDV, end diastolic volume; EDVI, indexed end diastolic volume; EF, ejection fraction; ESD, end systolic diameter: end systolic volume; ESVI, indexed end systolic volume; GLS, global longitudinal strain; HTN, arterial hypertension; IQR, interquartile range; IS, infero-septal; LV, left ventricular; PGD, primary graft dysfunction; PVD, peripheral vascular disease; RV, right ventricle; SV, stroke volume; SVI, stroke volume indexed; T2, relaxation time of myocardium (edema).

We investigated the association between structural and functional alterations and clinical outcomes, defined as a composite of cardiovascular hospitalization and mortality (Table 2). During the follow-up period, 14 events (a composite of cardiovascular hospitalizations and mortality), including overlapping occurrences, were identified. These events comprised 7 deaths and 11 hospitalizations, of which 10 were due to heart failure and 1 was due to stroke. The results revealed that, in univariate analysis, left ventricular (LV) stroke volume (LV SV) (HR 0.96, 95% CI 0.93-0.99, *P* = 0.01), indexed LV SV (HR 0.90, 95% CI 0.83-0.96, *P* = 0.01), LV ejection fraction (LV EF) (HR 0.90, 95% CI 0.85-0.95, *P* < 0.001), right ventricular (RV) stroke volume (RV SV) (HR 0.96, 95% CI 0.93-0.98, *P* = 0.003), indexed RV SV (HR 0.89, 95% CI 0.83-0.95, *P* < 0.001), RV ejection fraction (RV EF) (HR 0.91, 95% CI 0.85-0.97, *P* = 0.004), and LV scar size (HR 1.13, 95% CI 1.05-1.22, *P* = 0.002) were associated with an increased risk of cardiovascular hospitalization or mortality following orthotopic heart transplantation. After adjusting for age, gender, diabetes, hypertension, rejection treatment, ischemic time, and primary graft dysfunction, LV EF (HR 0.90, 95% CI 0.84-0.97, *P* = 0.004), indexed RV SV (HR 0.90, 95% CI 0.83-0.98, *P* = 0.015), and LV scar size (HR 1.17, 95% CI 1.02-1.33, *P* = 0.026) remained significant predictors of cardiovascular hospitalization or mortality.

**Table 2:** Univariate Analysis for Structural Myocardial Changes Detected by CMR Associated With Events

CMR parameters	Univariable		Multivariable	
	HR (95% CI)	*P*-values	HR (95% CI)	*P*-values
LV assessment				
LV GLS (%)	1.02 (0.89-1.17)	0.800	0.99 (0.80-1.21)	0.897
LV mass (g)	1.00 (0.98-1.01)	0.700	1.01 (0.99-1.02)	0.433
Indexed LV mass (g/m²)	1.00 (0.96-1.04)	0.960	1.02 (0.99-1.06)	0.197
LV SV (ml)	**0.96 (0.93-0.99)**	**0.010**	0.97 (0.93-1.00)	0.082
Indexed LV SV (ml/m²)	**0.90 (0.83-0.96)**	**0.010**	0.93 (0.86-1.00)	0.054
LV EF (%)	**0.90 (0.85-0.95)**	**<0.001**	**0.90 (0.84-0.97)**	**0.004**
LV wall thickness (anteroseptal)	0.20 (0.01-3.38)	0.270	0.06 (0.00-2.10)	0.122
LV wall thickness (inferolateral)	0.12 (0.01-2.37)	0.160	0.30 (0.01-9.18)	0.489
RV assessment				
RV GLS (%)	1.12 (0.96-1.30)	0.150	1.12 (0.93-1.35)	0.217
RV SV (ml)	**0.96 (0.93-0.98)**	**0.003**	0.97 (0.94-1.00)	0.063
Indexed RV SV (ml/m²)	**0.89 (0.83-0.95)**	**<0.001**	**0.90 (0.83-0.98)**	**0.015**
RV EF (%)	**0.91 (0.85-0.97)**	**0.004**	0.94 (0.87-1.03)	0.169
Tissue Characterization by CMR				
Global T2 (msec)	1.10 (0.99-1.21)	0.070	1.11 (0.98-1.26)	0.115
Global ECV (%)	0.90 (0.77-1.04)	0.149	0.98 (0.83-1.15)	0.767
LV Scar size (%)	**1.13 (1.05-1.22)**	**0.002**	**1.17 (1.02-1.33)**	**0.026**
Adjusted for age, gender, diabetes, hypertension, rejection treatment, ischemic time, and PGD.				

Values are in number (%) unless otherwise indicated.

CMR, cardiovascular magnetic resonance; ECV, extracellular volume; EF, ejection fraction; Global T2, relaxation time of myocardium (edema); GLS, global longitudinal strain; IQR, interquartile range; LV, left ventricular; PGD, primary graft dysfunction; RV, right ventricle; SV, stroke volume; SVI, stroke volume indexed.

**Figure 1:**
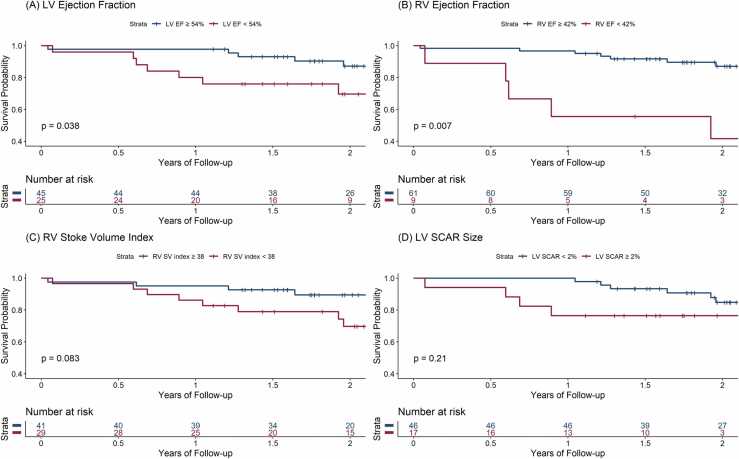
Kaplan-Meier survival curves were constructed to illustrate the cumulative incidence of the composite outcome (CV hospitalization and mortality), stratified by the ROC-derived cut-offs, with group differences assessed using the log-rank test. **(A)** Left Ventricle Ejection Fraction, **(B)** Right Ventricle Ejection Fraction, **(C)** Right Ventricle Stroke Volume Index, **(D)** Left Ventricle Scar Size.

The Kaplan-Meier survival curves, stratified by ROC-derived cut-offs, are shown for LV EF, indexed LV SV, RV EF, and indexed RV SV (Figure 1).

## Discussion

In this study, we examined CMR parameters in 70 heart transplant recipients, who were stratified into two groups based on their history of prior ACR before undergoing CMR. The results demonstrated lasting effects on the structure and function of both ventricles, even after rejection treatment. These effects were evidenced by decreased LV GLS and RV GLS, which serve as surrogates for early and subclinical cardiac dysfunction. In previous study,^12^ worse GLS has been associated with a long-term risk of death or major adverse cardiovascular events. The normal GLS value has not been well established in post-transplant patients. In some studies, GLS has been reported to be lower in transplant patients with normal ventricular function. However, the difference in GLS, potentially associated with the cumulative effect of ACR, could serve as a surrogate marker for early dysfunction. Further, impacts of prior ACR were observed in increased LV mass and LV wall thickness, which may reflect persistent edema or structural changes. These effects were not limited to structural alterations; we also observed functional and hemodynamic changes, as evidenced by decreased RV SV. These findings highlight that, even with treatment and resolution of ACR on biopsy, structural and functional myocardial abnormalities persist. This cumulative effect is observed in both ventricles but is more pronounced in the RV, as evidenced by its impact on RV hemodynamic changes. Recognizing these changes may help clinicians adopt a more proactive approach to monitoring and intervening, ultimately improving patient care and outcomes.

We also examined the CMR parameters associated with clinical outcomes. Structural and functional parameters of both the left and right ventricles, including left ventricular ejection fraction, right ventricular ejection fraction, left ventricular stroke volume (LV SV), RV SV, and left ventricular scar size, were all associated with clinical outcomes. After adjusting for relevant covariates, LV EF, RV SV, and LV scar size remained significant predictors of clinical outcomes. Scar tissue, already established as a marker of adverse outcomes,^4^ was further validated in this study.

To our knowledge, this is the first study to comprehensively examine the combined effects of functional, structural, and tissue parameters in heart transplant recipients, focusing on ACR's lasting impact on the myocardium using CMR. Previous studies have analyzed individual aspects such as strain,^1^, ^6^, ^13^ mapping,^4^ or LGE.^5^ This pilot study integrates early functional markers (eg, LV GLS, RV GLS), structural parameters (eg, LV mass, RV stroke volume), and tissue characteristics (eg, ECV, gadolinium T2 mapping, scar tissue), providing a holistic understanding of rejection's cumulative effects on the transplanted heart.

ACR is treated per center protocols, with rejection resolution confirmed by biopsy, which has limitations. CMR changes post-treatment provide insights into treatment success, the need for interventions, and risk stratification. The use of CMR in the post-transplant setting has been steadily increasing. Beyond its ability to assess cardiac structure and function, CMR offers superior tissue characterization, which may have important implications for risk stratification and the prediction of future adverse events. In the era of non-invasive methods such as donor-derived cell-free DNA, CMR offers an additional layer of valuable information.

Limitations include the single-center study, small sample size, and exclusion of ABMR. We did not consider the patients with ABMR; although the number of patients with significant ABMR in this population was very small, not considering ABMR may introduce bias by affecting the CMR parameters. Due to the single-center nature of the study, the generalizability of the findings may be limited. Larger, multicenter studies are needed to refine these findings.

## Conclusion

This pilot study integrates comprehensive CMR to assess the lasting effects of ACR on functional and structural damage in heart transplant recipients even after treatment. These CMR parameters correlate with clinical outcomes, highlighting their importance as critical markers.

## Declaration of Competing Interest

The authors declare that they have no known competing financial interests or personal relationships that could have appeared to influence the work reported in this article.
